# Recombinational micro-evolution of functionally different metallothionein promoter alleles from *Orchesella cincta*

**DOI:** 10.1186/1471-2148-7-88

**Published:** 2007-06-11

**Authors:** Thierry KS Janssens, Janine Mariën, Peter Cenijn, J Legler, Nico M van Straalen, Dick Roelofs

**Affiliations:** 1 Vrije Universiteit, Institute of Ecological Sciences, Department of Animal Ecology, De Boelelaan 1085, 1081 HV Amsterdam, the Netherlands; 2 Vrije Universiteit Amsterdam, Institute for Environmental Studies (IVM), de Boelelaan 1085, 1081 HV Amsterdam, the Netherlands

## Abstract

**Background:**

Metallothionein (*mt*) transcription is elevated in heavy metal tolerant field populations of *Orchesella cincta *(Collembola). This suggests that natural selection acts on transcriptional regulation of *mt *in springtails at sites where cadmium (Cd) levels in soil reach toxic values This study investigates the nature and the evolutionary origin of polymorphisms in the metallothionein promoter (*pmt*) and their functional significance for *mt *expression.

**Results:**

We sequenced approximately 1600 bp upstream the *mt *coding region by genome walking. Nine *pmt *alleles were discovered in NW-European populations. They differ in the number of some indels, consensus transcription factor binding sites and core promoter elements. Extensive recombination events between some of the alleles can be inferred from the alignment. A deviation from neutral expectations was detected in a cadmium tolerant population, pointing towards balancing selection on some promoter stretches. Luciferase constructs were made from the most abundant alleles, and responses to Cd, paraquat (oxidative stress inducer) and moulting hormone were studied in cell lines. By using paraquat we were able to dissect the effect of oxidative stress from the Cd specific effect, and extensive differences in *mt *induction levels between these two stressors were observed.

**Conclusion:**

The *pmt *alleles evolved by a number of recombination events, and exhibited differential inducibilities by Cd, paraquat and molting hormone. In a tolerant population from a metal contaminated site, promoter allele frequencies differed significantly from a reference site and nucleotide polymorphisms in some promoter stretches deviated from neutral expectations, revealing a signature of balancing selection. Our results suggest that the structural differences in the *Orchesella cincta *metallothionein promoter alleles contribute to the metallothionein -over-expresser phenotype in cadmium tolerant populations.

## Background

Transcriptional regulation plays an important role in the evolution of many phenotypes, especially when the phenotype correlates with expression level of a particular key gene. Transcriptional regulation is mostly controlled at the level of transcriptional initiation, i.e. the recruitment of transcription factors which determine the stability of the RNA polymerase II holoenzyme complex and hence the frequency of transcription initiation. Due to the modularity of transcription factor binding site clusters and the lack of reading frame constraint, promoters are more evolvable than coding regions [1,2]. A few point mutations in a *cis*-regulatory region can already confer functionally different phenotypes, even when the pattern does not deviate from neutral expectations[3,4]. Some examples of variation in regulatory loci conferring adaptive phenotypes are: the *LdhB *promoter of *Fundulus heteroclitus *[4-7], the *hsp70Ba *promoter of *Drosophila melanogaster *[8,9] the chalcone synthase promoter of *Arabidopsis thaliana *[3] and the *Cyp6g1 *promoter of *Drosophila melanogaster *[10]. In this paper we focus on transcriptional regulation of the *Orchesella cincta *metallothionein gene (*mt*), which is assumed to be involved in heavy metal tolerance.

Metallothioneins are low molecular weight metal-binding proteins with a high content of conserved cysteines within certain phylogenetic lineages and a lack of aromatic amino acids and histidine [11,12], although some invertebrate metallothioneins deviate from this pattern [13-15]. Metallothioneins are involved in essential metal homeostasis, metal detoxification, free radical scavenging, cell proliferation and apoptosis processes [16]. They are induced by several chemical and physical stresses [17], including free metal ions, altered redox status, oxidative stress and heat shock. The *Orchesella cincta *metallothionein (MT) [18] has a molecular weight of 7 kDa and consists of 77 amino acids. Almost all of the body burden of Cd is located in the gut epithelium and a major part of it is bound to MT [19,20]. It is suggested that the excretion of Cd from the animal occurs by a molting cycle regulated apoptotic process, by which the Cd-loaded midgut epithelium is shed [21,22]. Higher constitutive [23] and cadmium inducible [24]*mt *mRNA levels have been observed in populations from heavy metal contaminated sites, compared to populations from reference sites. Parent-offspring comparisons showed that Cd-induced expression of *mt *(h^2 ^= 0.48) is a heritable trait. Differences between expression level classes were linked to RFLP patterns of the *pmt *locus [25]. Although certain alleles of the *mt *coding sequence are linked to heavy metal pollution of the soil [26], it is rather unlikely that the heavy metal tolerance can be attributed to a single gene [27,28].

Inherited heavy metal tolerance has been associated with duplication events [29] and polymorphisms in the metallothionein coding sequence [26,30] The regulation of MT biosynthesis is mainly transcriptional and depends for the most part on *cis*-acting regulatory elements, such as the metal responsive element (MRE) which binds metal responsive transcription factor-1 (MTF-1), a Zn-finger protein, and the anti-oxidant responsive element (ARE) which recruits the nuclear erythroid derived related factor-2 (Nrf-2), a protein of the b-zip leucine zipper family [16,31]. However, this situation can not be generalized for all invertebrate phyla [32,33] Therefore, the induction of metallothionein can be considered as a concerted action of general, metal-specific and oxidative stress specific transcription factors.

In the past decades several studies on metallothionein promoters of invertebrates have been performed [34-42], although most mechanistic studies have been done on vertebrate model organisms, reviewed by [16,31,43]. One way to study the functionality of promoters is to fuse them to a quantitative reporter gene and analyze the induction *in vitro *in a host cell line. The comparison of allelic polymorphism in promoters by reporter assays has mainly been applied in medical biology, e.g. [44,45]. Only few studies in evolutionary ecology have compared alleles in this functional approach [5-7,46] and only one study compared the metal inducibility of metallothionein promoter alleles [47].

In the present study natural occurring allelic variation of the *Orchesella cincta *metallothionein promoter (*pmt *locus) is described. Following discovery of extensive variation in promoter sequence we formulated the following research question: "Are the *pmt *alleles observed in natural populations differentially induced by Cd, paraquat and 20-hydroxyecdysone (molting hormone) and can their induction be related to their different architecture?" Luciferase constructs were made and tested in an arthropod cell line for dose-dependent inducibilities. Paraquat is included in the experiment, because it generates reactive oxygen species in the electron transport chain [48] causing oxidative stress. This approach allows us to discriminate between the effects of Cd and oxidative stress separately. Finally, we tested if the *pmt *allele frequency distribution, based on nucleotide diversity measures, deviated from neutrality in a tolerant and sensitive population.

## Results

### General architecture of the *pmt *locus

**Figure 1:**
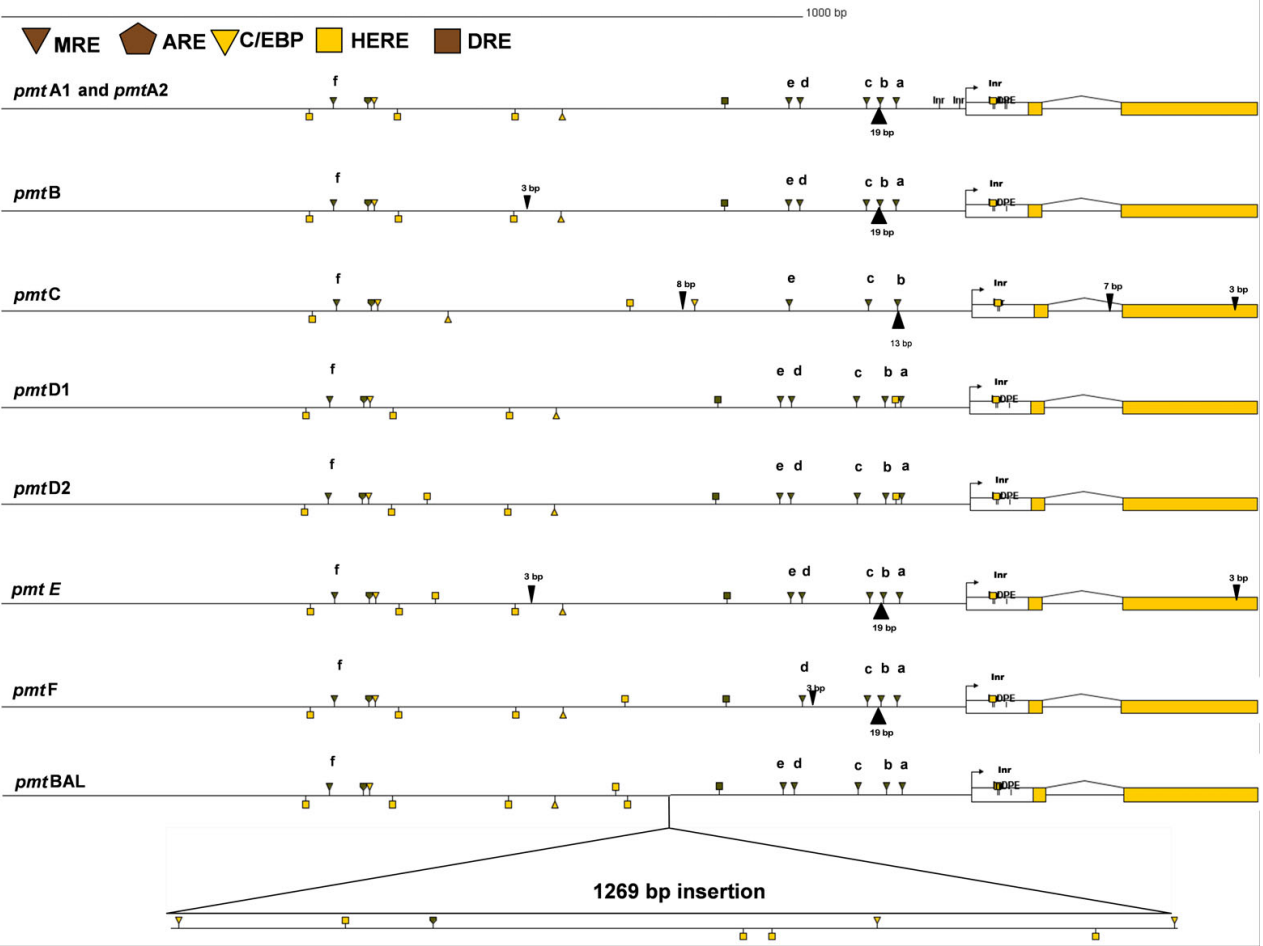
Architecture of the nine respective metallothionein promoter alleles (pmt). The respective putative transcription factor binding sites are represented as in the legend. Indels are indicated with black triangles. MRE, metal responsive element; ARE, anti-oxidant responsive element; DRE, DNA replication-related element; HERE, 20-hydroxyecdysone responsive element; Inr, initiator; DPE, downstream promoter element; C/EBP, CCAAT enhancer binding protein. The full sequence alignment is given in Additional File , 10.

Nine different alleles were identified in an alignment of 32 1500 bp promoter sequences (see additional files 1, 2, 3, 4, 5, 6, 7, 8, 9, 10). The consensus sequences of the respective alleles and the *O. villosa *were submitted to Genbank (DQ523588 to DQ523596, DQ641512, DQ523587 and EF106974). The general architecture of the nine promoter alleles is shown in Fig. 1, which also indicates positions of putative core promoter elements and transcription factor binding sites [16,31,49-51]. The number of the putative TFBS is summarized in Table 1 for each allele. The basal promoter consists of an initiator (Inr) consensus with an overlap of a 20-hydroxyecdysone responsive element (HERE). The *pmt*A allele contains two extra putative initiators. All the alleles, except *pmt*C, have a downstream promoter element (DPE) consensus downstream of their Inr. All *pmt *alleles contain MREs, which are all orientated in the sense direction. The proximal promoter, about 300 bp 5' from the Inr, contains most of the MREs. The alleles *pmtA1*, *pmt*A2, *pmt*B, *pmt*D1, *pmt*D2 and *pmt*BAL have five MREs in this region, named MRE-a to MRE-e. A number of indels in this MRE-rich region make this region variable. The MRE-a was apparently lost from the *pmt*C allele by a 13 bp deletion. A 19 bp deletion 5' of the MRE-b, relative to *pmt*C, *pmt*D1, *pmt*D2 and *pmt*BAL, characterizes *pmt*A, *pmt*B, *pmt*E and *pmt*F. This deletion affects the spatial position of the MREs. The *pmt*C and *pmt*F alleles have a point mutation which disrupts the consensus MRE sequence of MRE-d and MRE-e respectively. The *pmt*D1 and *pmt*D2 alleles share a HERE between MRE-a and MRE-b, by one point mutation. An AP-1 binding site consensus was only retrieved in the forward PCR primer D1-36F, and is not further discussed. This primer was developed after genome walking resulting in a clone that was apparently *pmt*A1. All alleles, except *pmt*C share a DNA replication-related element (DRE) [50].

**Table 1:** Summary of the occurrence of the respective putative transcription factor binding sites. a: The number of MREs is given as one in the enhancer region (in the vicinity of the ARE) plus the number in the proximal promoter. b: The number of HEREs is represented as the number in the region upstream of the initiator plus the one overlapping the initiator.

	Inr	DPE	MRE ^a^	ARE	C/EBP	DRE	HERE ^b^
Consensus	TCAKTY [49, 84]	RGWYV [84]	TGCRCNC [31]	TGA CNNNGC [31]	CCAAT [31]	TATCGATA [50]	KNTCANTNNMM [51]

*pmt*A	3	1	1+5	1	2	1	3+1
*pmt*B	1	1	1+5	1	2	1	3+1
*pmt*C	1	0	1+3	1	3	1	2+1
*pmt*D1	1	1	1+5	1	2	1	4+1
*pmt*D2	1	1	1+5	1	2	1	5+1
*pmt*E	1	1	1+5	1	2	1	4+1
*pmt*F	1	1	1+4	1	2	1	4+1
*pmt*BAL	1	1	1+5	2	5	1	9+1

A putative enhancer, ± 850 bp upstream from the Inr (when the 1269 bp insertion of *pmt*BAL is not taken into account), contains another MRE (MRE-f) and an anti-oxidant responsive element (ARE). Within this enhancer and towards the proximal promoter a number of HEREs and C/EBP (CCAAT enhancer binding protein binding site) binding sites are found scattered, which differ in number and position between the respective alleles. The 1269 bp indel in the *pmt*BAL allele is delineated at its edges (relative to the other alleles in the alignment) by two C/EBP binding sites. Furthermore this insertion contains 1 ARE, 3 HEREs and another C/EBP binding site.

### Similarities among *pmt *alleles and recombinant analyses

**Figure 2:**
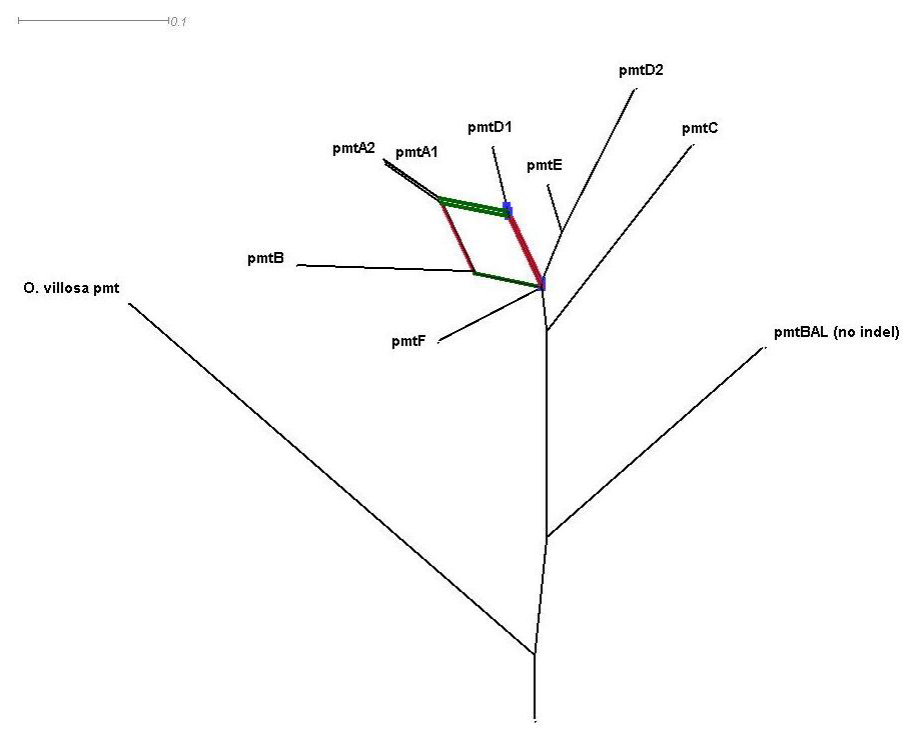
95% confidence reticulate network of the eight described *Orchesella cinca pmt *alleles with the *Orchesella villosa pmt *clone as an out-group, following 1000 replicate bootstraps in Splitstree v4 (uncorrected p for nucleotide substitution, NeighborNet to calculate the distance and Reticulate to calculate the splits). The colored parallel edges refer to the colors in the recombination analysis.

**Figure 3:**
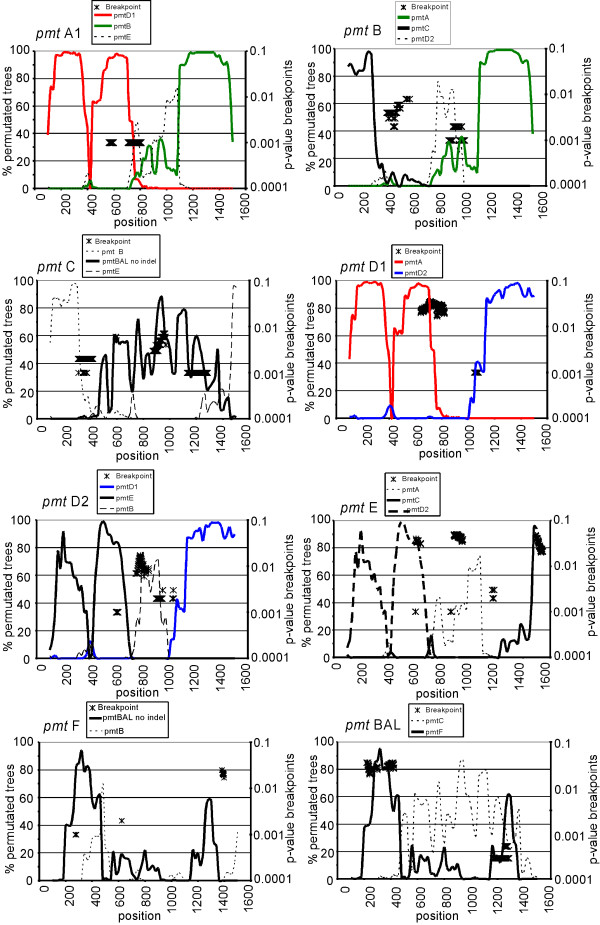
Recombination analysis of *Orchesella cincta *metallothionein promoter alleles. Bootscanning analysis representing the percentage of permutated trees (left axis) that did coincide between the respective *pmt *alleles in a sliding window approach (200 bp width, 20 bp step size, Kimura 2-parameter for nucleotide substitution) relative to the sequence position. Only the relationships which trespass the 70% threshold of the permutated trees are presented. On the right axis the p-values of the respective breakpoints are indicated. The colours refer to the parallel edges in the reticulate network (Fig. 2).

The phi test for recombination in the Splitstree4 program [52] found statistically significant evidence for recombination (p = 1.09 × 10^-13^). A bootstrap confidence network [52,53] based on a split decomposition analysis was developed representing the inferred recombination events in the *pmt *locus (Fig. 2). Split decomposition analysis addresses the problem of conflicting phylogenetic signals due to recombination which is not necessarily a branching or tree-like process. Parallel edges in the network represent evolutionary lineages of conflicting bifurcating trees. The parallel edges are presented in different colors in order to relate the events to results of the analysis below. The 1269 bp indel from the *pmt*BAL allele was omitted from these analyses. The evolution of the apparently ancestral alleles, *pmt*BAL, *pmt*C and *pmt*F, can be interpreted in a bifurcating pattern, whereas the more recent alleles *pmt*E, *pmt*D2, *pmt*D1, *pmt*B, pmtA2 and *pmt*A1 are consistent with a reticulate origin. The recombination analysis is presented in Fig. 3. Data below a bootscan threshold of 70% were omitted from the graphs. Breakpoints with their respective p-values from the Recco analysis, are plotted in the same graphs as a matter of convenience.

The *pmt*A1 and *pmt*A2 alleles consists of a ± 500 bp upstream block shared with *pmt*D1 and a ± 400 bp block shared with *pmt*B confirmed by very high bootstrap values. In between a slight similarity with *pmt*E was found. Recco confirmed this bootscan analysis and found a recombination breakpoint between the two dominant blocks. Allele *pmt*B contains a ± 150 bp upstream block related to *pmt*C and the ± 400 bp downstream block shared with *pmt*A as mentioned before. Both regions had recombination breakpoints at their respective 3' and 5' edges. The central part of the *pmt*B allele shares limited similarity with *pmt*D2. The *pmt*C allele has the shared ± 150 bp block with *pmt*B and a block shared with *pmt*E at the outer 3' end of the locus (the second exon of the *mt *gene), both confirmed by the Recco method, although not visible in the reticulate network. In between the two blocks bootscan analysis identified similarity with *pmt*BAL. Numerous recombination breakpoints were detected in this region, suggesting that this allele contains a recombination hotspot. The *pmt*D1 situation is very clearcut. This allele contains the ± 500 bp block shared with *pmt*A and a ± 400 bp 3' block shared with *pmt*D2, confirmed by the Recco method. Both regions have numerous putative recombination breakpoints at their edges. The *pmt*D2 allele shares a ± 500 bp upstream block with *pmt*E and the downstream block with *pmt*D1 mentioned before, confirmed by the Recco method. In between the respective blocks a smaller ± 100 bp block similar to *pmt*B, surrounded by recombination breakpoints, was found. The *pmt*F allele has a ± 100 bp upstream block shared with *pmt*BAL immediately followed by a very small block shared with *pmt*B. Only the former block was confirmed by Recco. The *pmt*BAL situation was rather contradictory, although some recombination breakpoints provided by Recco confirmed the bootscan similarity profile with *pmt*F. The limited similarity with *pmt*C was not supported by Recco. *pmt*E consists of the clear cut upstream ± 500 bp block shared with *pmt*D2 and the shared second exon with *pmt*C. On the other hand, the central part showed some above threshold similarity with *pmt*A1. Again numerous putative recombination breakpoints, at the edges of some shared blocks, were detected by Recco.

The deep trenches in the respective recombined blocks represent conserved modules, e.g. the one at position ± 400 bp is the region containing the ARE and the distant MRE.

### Functional analysis

The functional significance of *pmt *variation was assessed by evaluating the effect of the different promoters on gene expression. Luciferase reporter assays of pGL3-*pmt *constructs were performed in *Drosophila *S2 cell line. No induction of the empty vector pGL3Basic neither the normalization vector *pAc5.1/V5-His/lacZ *was observed (data not shown), implying that the observed induction of the pGL3-*pmt *constructs are exclusively due to the interaction of the transcription factors from the host cells with the *mt *promoters in the luciferase constructs. As a positive control for ecdysone treatment the 20-E exposure was also performed on cells transfected with the construct *pEcRhspluc *[54], a highly 20-E inducible luciferase construct (results not shown). Indeed, a high induction level was observed, comparable to Poels et al (2004) [45].

**Figure 4:**
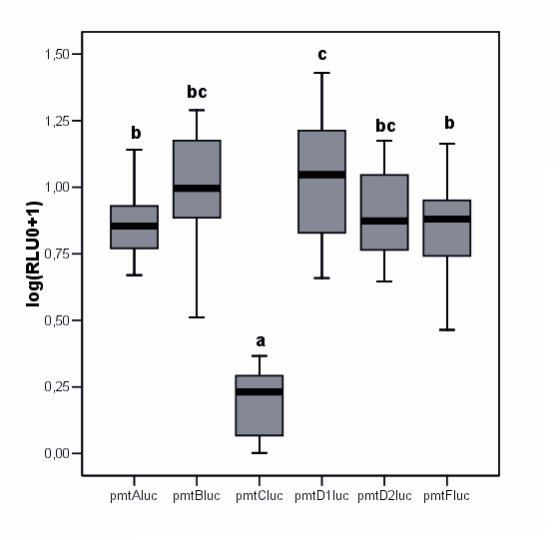
Boxplot representing the log transformed basal expression RLU values. One Way ANOVA; F = 71.337 and p = 0.000. A Tukey post-hoc test revealed significance groups, represented by letters.

Basal luciferase expression data are presented in Fig. 4. Highly significant differences in basal expression were detected between the luciferase constructs from the different alleles, following a One Way ANOVA test, with the Tukey HSD post-hoc test. The basal expression from the *pmt*C*luc *construct hardly deviated from the empty vector pGL3Basic (data not shown) and was an order of magnitude lower than the basal expression values of the other constructs. The *pmt*D1luc had a higher basal expression than the *pmt*A1, *pmt*F and the *pmt*C constructs.

**Figure 5:**
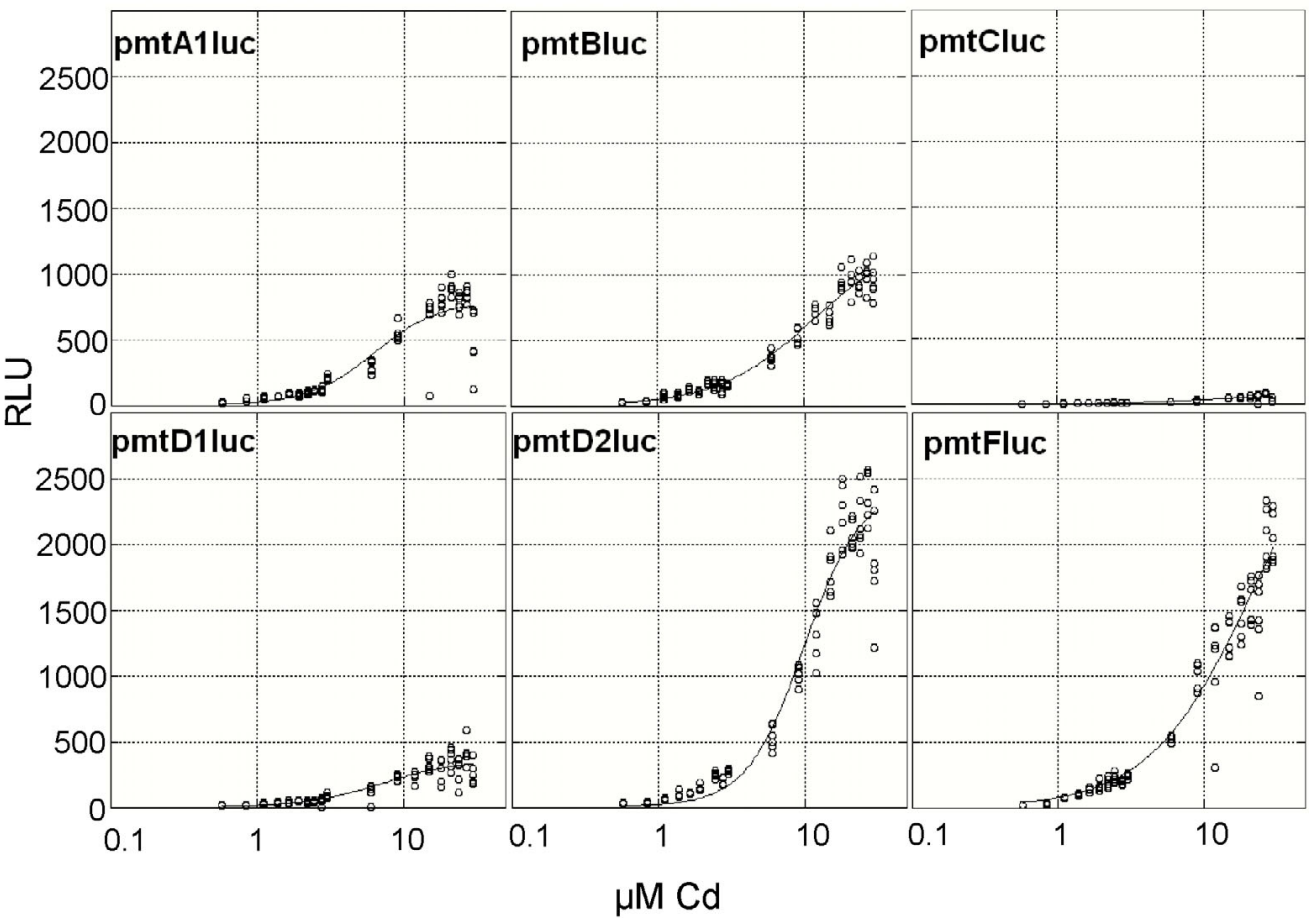
Dose response relationships of the six luciferase constructs. On the Y-axis the β-galactosidase normalized relative luciferase units (RLU) are presented. On the X-axis the exposure concentrations of cadmium (Cd) are indicated.

**Figure 6:**
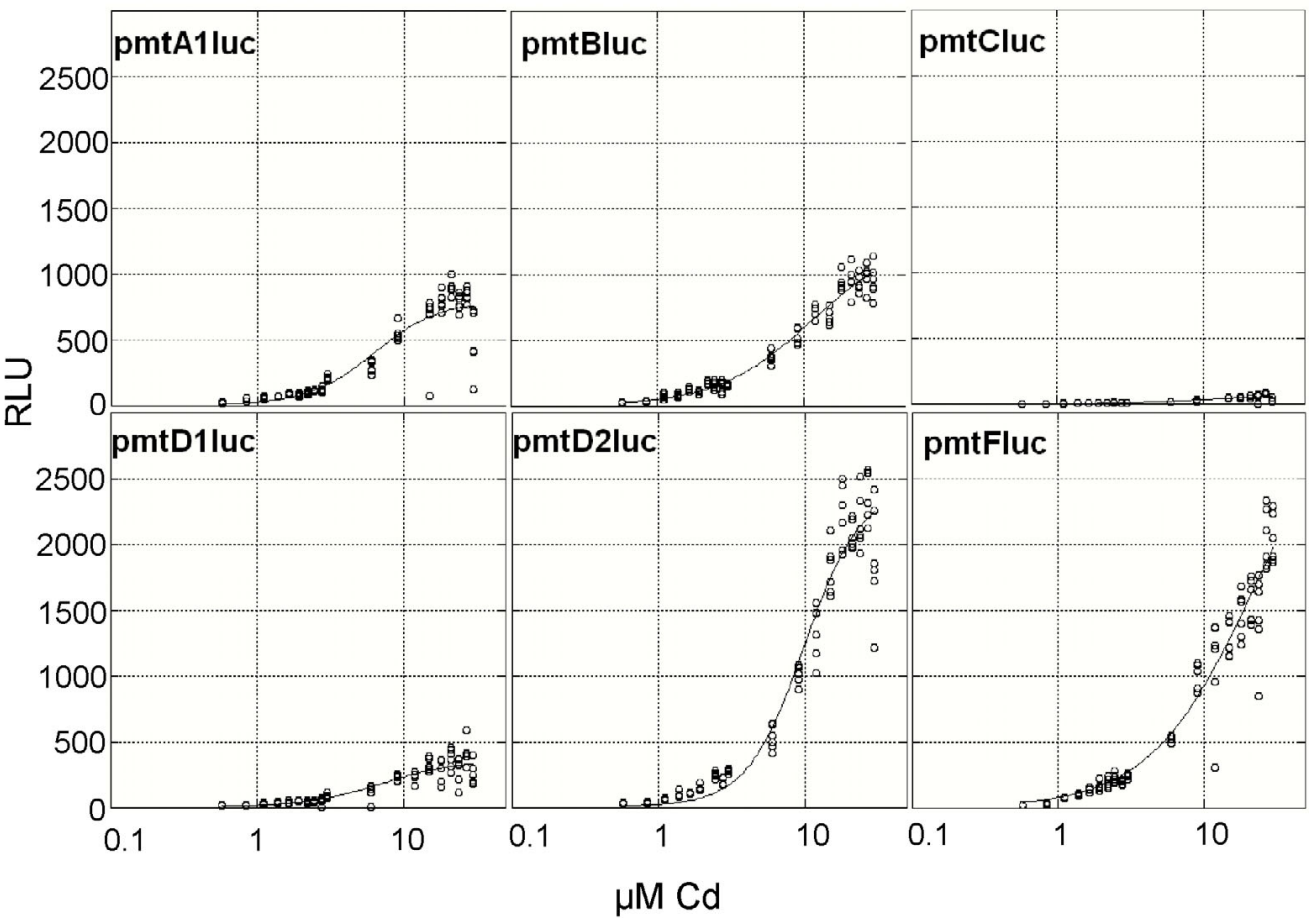
Dose response relationships of the six luciferase constructs. On the Y-axis the β-galactosidase normalized relative luciferase units (RLU) are presented. On the X-axis the exposure concentrations of paraquat are indicated.

**Figure 7:**
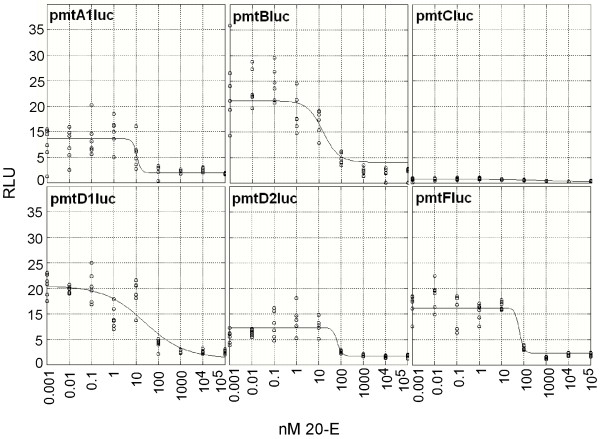
Dose response relationships of the six luciferase constructs. On the Y-axis the β-galactosidase normalized relative luciferase units (RLU) are presented. On the X-axis the exposure concentrations of 20-hydroxyecdysone (20-E) are indicated.

**Figure 8:**
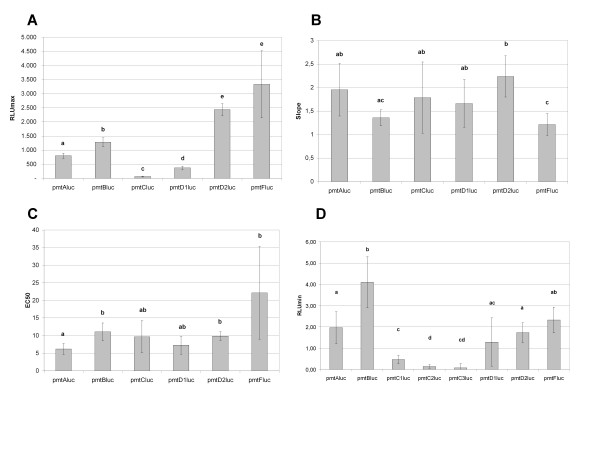
A: RLU_max _estimates from the cadmium (Cd) exposure data, B: Slope estimates from the Cd exposure data, C: EC_50 _estimates from the Cd exposure data D: RLU_min _from the 20-E exposure data.

The dose response graphs from the Cd, paraquat and 20-E exposures and their estimated parameters are given in Figs. 5, 6, 7 and 8 respectively (see Additional File , 11 for a numerical summary of the curve fit data). It appeared that all constructs were susceptible to Cd. The RLU_max _estimates of *pmt*D2*luc *and *pmt*F*luc *Cd exposures did not differ and were the highest observed. Exposure of the other constructs to Cd resulted in significantly different RLU_max _values. The pmtC*luc *constructs, with the lowest RLU_max _were the least inducible. The slope of *pmt*D2*luc *in the Cd exposure was steeper than *pmt*B*luc *and *pmt*F*luc*, implying a quicker inducibility. The most sensitive construct to induction by Cd, represented by the lowest EC_50_, was *pmt*A1*luc*. It differed significantly from the least sensitive constructs *pmt*B*luc*, *pmt*D2*luc *and *pmt*F*luc*.

The *pmt*C*luc *construct did not show a significant Pearson correlation with paraquat concentration (p > 0.05) and no model fit was possible. No significant differences in the estimated parameters of the paraquat exposure were found in the responses of the inducible constructs. The 20-E exposure data revealed an inhibition of every construct. The estimated values for the parameters were abandoned, because of the wide associated 95% confidence intervals, therefore only the estimates for the RLU_min _value are represented. It appeared that the *pmt*B*luc *construct was the least inhibited at the maximum exposure concentrations of 0.1 mM 20-E, compared to the other constructs.

At the lower range of the 20-E exposure a slight induction was observed in all constructs. We tested the RLU estimated from the inducing concentration range relativeto the unexposed control in a One Way ANOVA approach (see Table 2). The *pmt*B*luc *and *pmt*D2*luc *constructs were both significantly induced at respectively 0.1 nM and 1 nM 20-E exposure.

**Table 2:** One Way ANOVA test comparing the measured RLU values at the control and at the putative inducing 20-E concentration. F-ratios and p-values are given.

Construct	[20-E] (nM)	F-ratio	p-value
*pmt*A*luc*	0.1	0.121	0.739
*pmt*B*luc*	0.1	10.320	**0.009**
*pmt*C*luc*	1	1.783	0.211
*pmt*D1*luc*	0.1	0.256	0.624
*pmt*D2*luc*	1	7.812	**0.019**
*pmt*F*luc*	0.01	4.598	0.058

### Allele frequencies in field populations

The occurrence of the *pmt *alleles was assessed in *O. cincta *populations from a clean reference site (Roggebotzand) and a metal-polluted site (Plombières). These data are summarized in Table 3, together with information on soil metal concentrations and several indices of Cd tolerance in the two populations obtained from earlier work (Cd excretion, growth reduction and *mt *expression). The insert of the *pmt*BAL allele is omitted from this analysis.

**Table 3:** Background information of the sampled populations and summary of the molecular evolutionary analysis in DnaSP.

		Plombières	Roggebotzand
[Cd]_tot _soil (mg/kg) (Janssens, unpublished)		30.45 ± 14.82	0.17 ± 0.05
Allele frequencies per population (%) (Janssens, unpublished)	*pmt*A1	32.9	47.0
	*pmt*A2	7.6	15.7
	*pmt*B	24.7	10.4
	*pmt*C	11.4	2.2
	*pmt*D1	10.1	13.4
	*pmt*D2	8.9	1.5
	*pmt*E	0.6	6.0
	*pmt*F	1.9	2.9
	*pmt*BAL	1.9	0.7
	N	79	67
Sample size (number of alleles)		158 (9)	134 (9)
S (ç)		201(214)	201 (214)
H_d_		0.798 ± 0.017	0.726 ± 0.032
π		0.02634 ± 0.00159	0.01491 ± 0.00162
Tajima's D		0.17187 NS	-1.3781, NS
Fu and Li's D		2.88149, p < 0.02**	-0.75998, NS
Fu and Li's D*		2.77359, p < 0.02 **	-2.13596, NS
Average constitutive *mt *expression. MNE relative to β-actin (Roelofs, unpublished)		0.52 ± 0.18	0.04 ± 0.00
Induced (1 μmole Cd/g food) *mt *expression. MNE relative to β-actin (Roelofs, unpublished)		2.63 ± 0.57	1.18 ± 0.66
Mean Cd excretion efficiency per moult (Posthuma 1993)		45%	38%

Total cadmium (Cd) content of the soil; allele frequencies of the *pmt *alleles; S, total number of variable (segregating) sites; η, total number of mutations; Hd, haplotype diversity ± SD; π, nucleotide diversity per site ± SD; Tajima's D, Fu and Li's D and Fu and Li's D*, mean normalized expression, p-value of the G-test performed on the reconstituted dataset (df = 8), MNE, mean normalized expression at mRNA level; Cd excretion efficiency; percentage of Cd-induced growth reduction.

**Figure 9:**
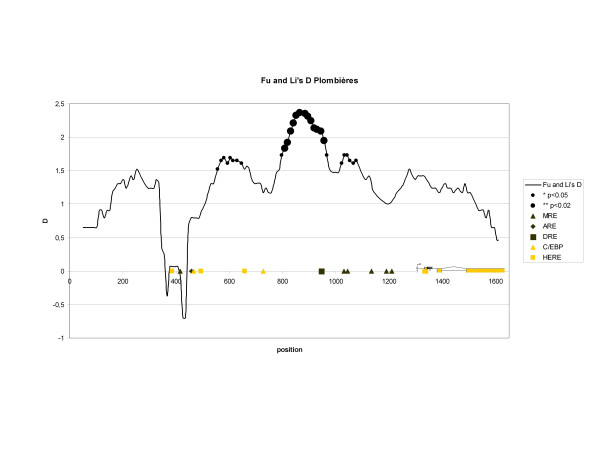
Sliding window analysis of the Fu and Li's D statistic on the reconstituted dataset of the Plombières population. A step size of 10 bp and a window length of 100 bp were applied. Significant deviations from neutral expectations are represented with ● and ● for p-values < 0.05 and 0.02 respectively. A cartoon of the general architecture of the *pmt *locus is provided with the putative transcription factor binding sites.

Pollution by heavy metals in the abandoned Pb-Zn mine of Plombières dates back to the Middle Ages and has proceeded until the beginning of the 20^th ^century [55]. This is reflected by the almost 200-fold higher total Cd content of this soil compared to the clean site in Roggebotzand. This latter site is located on reclaimed land, which fell dry in 1968. A G-test for differences of allele frequencies between both populations was highly significant (p < 0.001). The frequencies of *pmt*A1 and *pmt*A2 are relatively low in the population from the mining site Plombières, while the *pmt*C, *pmt*B and *pmt*D2 alleles are more represented compared to the situation in the reference Roggebotzand population. In addition, a higher nucleotide, haplotype and nucleotide diversity was observed in the Plombières population. When calculating the Tajima's D test, no deviation from neutral expectations was observed. The Fu and Li's tests, on the other hand, did show a significant departure from neutrality in the Plombières population, both with and without taking the out-group into account (D and D* respectively). Positive values of D and D* suggest the presence of an excess of intermediate frequency variants in the sample, which means that balancing selection is acting. When we calculated the Fu and Li's D in a sliding window approach, the promoter stretches on which balancing selection is taking place can be identified (see Fig 9). The spectrum of Fu and Li's D is aligned to a graph of the general architecture of the *pmt *locus. Non-significant, but negative D values are observed in the vicinity of the putative enhancer. This suggests that this stretch is under positive selection. Truly significant deviations from neutrality, with positive D values, are detected in the region between the two C/EBP binding sites, upstream from the DRE and in the vicinity of the MRE-d and MRE-e.

## Discussion

From our data we have very good evidence that allelic diversity at the *pmt *locus has evolved by extensive recombination events, although we do not have knowledge about the genetic mechanism, e.g. crossing-over or gene conversion. The patterns in the splits decomposition network could somehow be linked to the results of the recombination analysis. The most important recombination blocks, which became evident in the bootscanning and Recco methods, were visible in the network. Especially the more recent alleles, *pmt*A1, *pmt*B, *pmt*D1, *pmt*D2 and *pmt*E revealed clear-cut signals of recombination among each other. Recombination between the older alleles, *pmt*BAL, *pmt*F and *pmt*C, were not detected at all in the Recco and the Splitstree method Reasons for this could be that the parental alleles were not sampled or that former recombination events could be hidden behind the mutational load. Because the Recco method takes the minimization of recombination and mutation costs into account, instead of the tree-like model in the bootscanning approach, it detects recombination false positives to a lesser extent [56]. The absence of reticulations in the edges of these alleles in the 95% confidence reticulate network, points towards less conflicting bifurcations (Fig. 2). These may be caused by the accumulation of mutations following past recombination events.

The numerous recombination breakpoints at the *pmt *locus can be explained by the fact that transcriptionally active chromatin with recruited transcription factors is hypersensitive to recombination initiation [57]. Not transcription *in se *but the recruitment of recombination machinery by environmentally activated transcription factors following chromatin remodeling causes recombination events. For example, in fission yeast, phosphorylation by the stress-activated protein kinase from the MAPK pathway increases the affinity of the transcription factor ATF1. PCR1 for a cAMP-responsive element-like (CRE-like) DNA sequence and remodels the chromatin [58]. This process and the respective CRE-like sequence are associated with a recombination hotspot in fission yeast [59]. In every *pmt *allele two CRE core sequences, CGTCA [60] were found. Because of the importance of the composition of the flanking sequences of the CRE core sequence and the large number of CREB proteins, these data are not further discussed, although it may be of importance for transcriptional regulation and initiation of recombination at the *Orchesella cincta pmt *locus.

The 1269 bp insertion in the *pmt*BAL allele, which does contain some relevant putative transcription factor binding sites, is suggested to be a possibly recombined or duplicated region from another promoter. Similar events, where promoter regions were swapped between loci, have been described before [46].

The number of MREs in stress gene promoters in general [61] and metallothionein promoters in particular varies extensively. The same applies to their sequence and their spatial context. However, three MRE's (MRE-b, MRE_c and MRE-e) were conserved in sequence (TGCACAC) between *O. cincta *and the out-group species *O. villosa*. Previous studies revealed that the proximal MRE cluster in metal-responsive promoters is necessary [62] and MREs in the proximal promoter region need to work cooperatively for the full inductive capacity [36,43,63]. The most proximal MRE to the transcription start (MRE-a), has been shown to be most contributing to induction by Zn and Cd of the human *hmt-IIA *promoter [64] and cooperates with more upstream MREs for the complete heavy metal induction [65].

The induction by paraquat was several orders of magnitude lower than the induction by Cd, which possibly reflects that not all the MREs are involved. In a study by [66] three different oxidative stressors elicited a twofold response of the rainbow trout MT-B promoter luciferase constructs, comparable to our study with paraquat. Beside the ARE and the MRE-a it was suggested that ARE half-sites (TGAC) are as well responsible for oxidative stress inducibility [66].

The reporter assays show that allele C is hardly inducible by Cd and oxidative stress (Fig. 5) and shows no basal transcription level above background (data not shown). This may be explained by the deletion of the MRE-a in this allele as follows. During exposure to Cd and paraquat, *trans*-activation in non-mammalian metallothionein promoters can occur by interaction of an enhancer (containing MREs and/or ARE) with the MRE-a and other proximal MREs [43,63,66-69]. The induction of *pmt*C by Cd is low and completely abolished by paraquat possibly due to the incapability of *trans*-activation between the MRE-a (lacking in the C allele) and the distal enhancer, directly or in combination with CCAAT/enhancer binding proteins [70,71]. Binding sites for CCAAT/enhancer binding proteins were detected in all alleles of the *pmt *locus. Also, the MRE-a is often important in determining basal expression levels [43,64-66]. Deletion of MRE-a in *pmt *C can therefore explain the low basal expression of this allele. Finally the importance of MRE-a is reflected in the highly conserved core sequence: *O. cincta *MRE-a has the same core sequence as *Onchorhynchus mykiss*, *Strongylocentrotus purpuratus *and *Drosophila *metallothionein promoters.

There may be an alternative explanation for the very low basal expression levels of *pmt*C as well as low stress induction, related to the absence of DRE. The DRE [50] is an element found in DNA replication related genes, as well as in stress responsive genes (a.o. glutathione-S-transferase, catalase). This element coordinates the cell cycle specific expression of metallothionein. Since *pmt*C lacks this element (disruption of the consensus by a point mutation) it may contribute to the low inducibility and basal expression.

We realize that we tested *O. cincta *derived promoters in a *Drosophila *genetic background. Thus, the observed levels may not reflect the levels *in vivo *due to absence of *O. cincta *specific transcription factors or altered binding specificity of *Drosophila *transcription factors to *O. cincta *specific transcription factor binding sites. Reporter assays are sometimes unable to generate the tissue, temporal and species specific transcriptional regulation [72-75]. For instance, aberrant expression levels have been observed when expression levels of 12 orthologous genes of human and chimpanzee were compared in cell lines from different human tissue origin [76] when compared to their *in vivo *expression levels. In contrast with that, Crawford *et al *[7] obtained consistent results using two unrelated fish cell lines (rainbow trout hepatoma cells and salmon cardiac cells) to study transcriptional activity of killifish lactate dehydrogenase-B promoters.

The general pattern of 20-E exposure is the inhibition of the metallothionein promoter. This probably occurs because of the overlapping HERE and Inr. The moderate inducibility of *pmt*B and *pmt*D2, at respectively 0.1 and 1 nM 20-E, can not be explained straightforward, although *pmt*D2 has a larger number of HEREs (5+1, See Table 1). The spatial and sequence context could be the reason for their inducibility. In the firebrat *Thermobia domestica *20-E equivalents peak to 5 μM during apolysis [77] falling back to the basal concentration of 80 nM, indicating that the order of magnitude of our exposure range was well within the physiologically relevant range. These data suggest that the metallothionein expression is switched off during ecdysone-induced apoptosis of the gut epithelium, when the onset of new cuticle formation is set.

Our small-scale population genetic comparison between populations from the clean site in Roggebotzand and the polluted one in Plombières reflected a positive sign of balancing selection at Plombières, because of the significant positive Fu and Li's D values, higher nucleotide diversity per site, and higher haplotype diversity in the latter population. A study by Timmermans *et al*. [26] revealed selection on certain alleles of the *O. cincta mt *coding sequence by heavy metal content in the soil. However, no signatures of any selection were detected in the amino acid sequence (d_n_/d_s_, Fisher's exact test) or in the nucleotide composition (Tajima's D). The population at Plombières is characterized by relatively high frequencies of *pmt*D2 and *pmt*B, which is in accordance with their greater inducibility compared to the most common *pmt*A1 allele. This suggests a fitness advantage to phenotypes with high metallothionein expression at polluted sites [23]. On the other hand, the Plombières population also has a relatively high frequency of the less responsive *pmt*C allele, This is consistent with the signature of balancing selection detected by Fu and Li's D values and can be understood if there are not only advantages in terms of metal tolerance but also fitness costs associated with high *mt *expression. Further work is necessary to elucidate the precise relationship between fitness and *pmt *genotype.

## Conclusion

The *Orchesella cincta *metallothionein promoter contains a high degree of polymorphism, reflected in the different number and spacing of consensus transcription factor binding sites involved in relevant regulatory processes, such as heavy metal, oxidative stress induction and the regulation by the molting cycle.

In general, the evolution of transcriptional regulation can occur by stabilizing selection by which erosive mutations in TFBS and the emergence of new ones are compensating each other. Evolution due to transcriptional regulation has been reported in macro-evolutionary processes, e.g. the even-skipped-2 enhancer of *Drosophila *species [78-80]. Alternatively, micro-evolutionary processes rather occur by point mutations in (putative) TFBS [3,6,7]. Here, we provide evidence of extensive recombination, reshuffling the nucleotide variation of insertion, deletions and point mutations in the micro-evolution of a regulatory locus.

Since induction of metallothionein is suggested to be associated with a metal-tolerant phenotype [24,25] the difference in inducibility between the *pmt *alleles provides a scope for natural selection in field populations. The deviations from neutral expectations in the cadmium tolerant population from Plombières support this suggestion. Future approaches will be the detection of DNA-binding activity of selected transcription factors, measure the *in vivo *transcription by real-time Q-PCR, and screen field populations for their respective *pmt *allele frequencies.

## Methods

### DNA purification

DNA of individual animals was purified by the SV genomic DNA purification system (Promega Corporation). The maxiprep used for the genome walking procedure was done by a modified CTAB extraction method [81] on 100 adult individuals.

### Genome Walking and PCR

Different aliquots of 10 μg of genomic DNA of *Orchesella cincta *(laboratory culture) and *Orchesella villosa *(Belcaro, Italy) were digested overnight with 5 to 10 U different blunt cutting restriction enzymes, *Dra*I, *Eco*RV, *Hinc*II, *Hpa*I, *Sca*I, *Sma*I and *Stu*I. These digests were cleaned up by phenol chloroform extraction, followed by ethanol precipitation and consequently ligated overnight at 4°C to the adapter originating from the hybridized adapters 1 and 1A (see Additional File , 12).

Based on the sequences resulting from the Universal fast walking method [82] on the metallothionein promoter, (Mariën and Roelofs, unpubl.), a nested reverse primer pair, (R34 and R130, see Additional File , 12) in the proximal promoter was designed. A nested gradient PCR approach was conducted with above mentioned primers to each of the differentially digested aliquots. The same approach was done in two steps for the *O. villosa *promoter with respectively the primer pairs (Ovimt210R and Ovimt233R) (Spinsanti, unpubl.) and the resulting primer pair (Ovipmt and Ovipmtnested).

In the resulting sequences for *O. cincta *and *O. villosa*, the respective primers, D1-36F and OvipmtfarF were developed on the 5' end. The metallothionein promoter was amplified by PCR (T_ann _= 55°C) with the primer combination D1-36F and MT-265R [25] and OvipmtfarF and Ovimt233R for *O. cincta *and *O. villosa *respectively. The reverse primer hybridizes in the second exon of the *mt *gene, and consequently allows the alignment of the previously described *Orchesella cincta mt *sequences (Timmermans *et al*. in press) [25]) to the resulting *Orchesella cincta pmt *sequences.

### DNA-Sequencing

32 *pmt *fragments, originating from various populations (see Additional File , 13), with previously known proximal promoter SSCP genotype or with deviating RFLP patterns (Janssens, unpublished) were amplified with the D1-36F an MT265R primer pair. Resulting PCR fragments were ligated in the pGEM-T vector (Promega Corporation) and cloned in JM109 or XL1-Blue competent cells by respectively heat-shock or electroporation procedures. Plasmid purification was executed with the SV Minipep System (Promega Corporation). The clones were sequenced with Big Dye V 1.1 (ABI) on a ABI 3100 capillary sequencer, analyzed with Vector NTI Software 10.0.1 (Invitrogen) and aligned to the *mt *alleles previously described by Timmermans et al. (pers. comm.). The alignments served to make a consensus for every allele, and are provided as Additional Files 2, 3, 4, 5, 6, 7, 8, 9. One single *O. villosa pmt *sequence (Zelzate, Belgium) was cloned and sequenced to serve as out-group in phylogenetic analysis.

### Transcription Factor Binding Site Analysis

Because of the lack of functional studies on this promoter, the descriptive work of the observed sequence variation was restricted to the identification of consensus binding sites available in the literature by using the program Genepalette 1.2 [83]. The core promoter structure was analyzed by using consensus sequences from the literature [49,83,84]. Consensus binding sites for transcription factors known from metallothionein induction [16,31] cell cycle regulated and stress genes [50] and molting cycle regulated transcription processes [51] were included in the analysis.

### Luciferase reporter assay

From six of the *O. cincta pmt *alleles a luciferase reporter construct was made by PCR on the respective minipreps with the primers, D136-F*Kpn*I and MT-73R*Xho*I, containing restriction sites for these respective enzymes. The clones on which these luciferase reporter constructs were made are summarized in Additional File , 13 section. The fragments were ligated in *Kpn*I and *Xho*I double-digested pGL3Basic luciferase vector (Promega Corporation) and cloned in XL1-Blue by electroporation. As an internal control for transfection efficiency and number of cells, a β-galactosidase reporter plasmid with a constituve actin promoter of *Drosophila melanogaster *(pAc5.1/V5-His/lacZ (Invitrogen), was used. Purification of transfection grade and endotoxin-free plasmid was done with the Nucleobond PC 500 EF kit (Macherey-Nagel)

*Drosophila *S2 cells (Gibco) were grown at 28°C in Schneiders *Drosophila *Medium (revised) (Gibco) containing 15% fetal calf serum (Gibco). On the first day cells were plated out in 96 wells plates at approximately 25000 cells/150 μl and grown for 24 hours at 28°C. The second day every well was transfected using a calcium phosphate precipitation method [85] with 0.72 μg of DNA in 15 μl. The DNA used for transfection was a 1/1 ratio of the respective luciferase constructs with the pAc5.1/V5-His/lacZ. Following overnight incubation the medium was removed and replaced by spiked exposure media. Exposure ranges for Cd, paraquat and 20-hydroxyecdysone (20-E) were 0–30 μM, 0–1800 μM and 0–100 μM respectively. Every exposure was executed in six wells. The basal expression was measured in hexaplicate in four independent experiments.

Thousand fold stock solutions of the respective exposure concentrations were filter sterilized (0.2 μm) and stored at 4°C. Cadmium and paraquat were dissolved in water whereas 20-E was dissolved in DMSO. The 20-E stock in DMSO was not filter sterilized. Schneider's *Drosophila* Medium (revised) (Gibco), containing 5% fetal calf serum (Gibco), was spiked with the respective stock solution to achieve the required final concentrations.

The exposure with Cd and 20-E was performed during 24 hours and the paraquat exposure was limited to 6 hours due to cytotoxicity. After the exposure period the cells were lysed in 100 μl lysis buffer containing (25 mM Tris, 2 mM dithiothreitol, 2 mM trans-1,2-diaminocyclohexane-N,N,N9,N9-tetraacetic acid monohydrate, 10% glycerol, and 1% Triton^® ^X-100 [Sigma-Aldrich, Steinheim, Germany] in demineralized water, pH 7.8 overall buffer).

A 50 μl aliquot was used to measure luciferase activity using 100 μl glowmix, (20 mM tricine, 1.07 mM C4H2Mg5O14, 2.6 mM MgSO4, 0.1 mM ethylenediamine-N,N,N9,N9-tetraacetic acid, 33.3 mM dithiothreitol, 0.27 mM coenzyme A, 0.46 mM luciferine, and 0.53 mM adenosine-59-triphosphate in demineralized water), on a LucyII luminometer (Anthos Labtec instruments). Beta-galactosidase activity was measured in a 50 μl lysis aliquot, diluted in 100 μl lysis buffer and 90 μl of ONPG-mix. The ONPG mix consisted of 24 μl of 10xZ buffer (60 mM Na2HPO4, 40 mM NaH2PO4, 10 mM KCl and 2 mM MgSO4), 66 μl water, 0.1 μl β-mercaptoethanol and 0.16 mg 2-nitrophenyl β-D-galactopyranoside (ONPG). The reactions were incubated during one hour at 28°C and subsequently the absorbance at 420 nm was measured on a Spectramax 340pc (Molecular Devices) spectrophotometer.

Firstly, luciferase signals in each well were normalized with an internal luciferase standard, in order to standardize the readings between different plates. Secondly, the normalizations for the number of cells, and the transfection efficiency, were done by dividing the latter values by A_420 _values from the ONPG measurement. The basal expression values of the constructs and the pGL3Basic vector were measured in four replicate experiments, six wells per experiment.

### Data analysis

DNA sequences were processed and aligned in Vector NTI version 10.0.1 (Invitrogen). Recombination events, which took place at this locus, make the construction of a bifurcating tree less relevant. Therefore, the Splitstree4 software [52] was used to construct a reticulate network to represent evolutionary relationships between the respective alleles. The assumptions under which the network was constructed are: uncorrected P for nucleotide substitution, NeighborNet to calculate the distances and the reticulate method to treat the splits. Gaps, constant and non-parsimonous positions were omitted from the analysis. Bootstrap analysis was performed on one thousand replicates and subsequently a 95% confidence network was constructed.

Recombination sites were detected by using two methods. The first method we used relies on the bootscan method implemented in the program Simplot [86]. It constructs replicate trees in a sliding window approach. The general accepted threshold level for the detection of a recombination is a clustering in 70% of the permutated trees in a certain window. Thousand replicate neighbor-joining trees were made by using the following parameters, window size 200 bp, step size 20 bp and the Kimura 2-parameter as a model to estimate nucleotide substitution. Alternatively, a non-phylogenetic method, applying dynamic programming that minimizes the mutation and recombination cost between sequences was used. This method is implemented in the software Recco [56]. The parameter α, representing the ratio of mutation cost to recombination cost was set to 0.2, the methods to calculate mutation and recombination costs were respectively Hamming and Delta Dirac.

The population genetic data were achieved by RFLP analysis of the amplified *pmt *fragments by PCR (Janssens unpublished). A reconstituted dataset was made by pasting the consensus for every allele the number of times it was observed in the sample, according to Timmmermans *et al*., in press These datasets were aligned with each other and an alignment with the out-group *Orchesella villosa pmt *sequence was provided. Molecular diversity indices and deviations from the neutral theory (Tajima's D, Fu and Li's D and D*) were calculated using DnaSP v4.10 [87].

The basal expression RLU data were log(x+1) transformed to approach normality of the data, A One Way ANOVA with p < 0.05 was executed. The LSD post-hoc test was performed to discriminate significance groups.

Initial curve-fitting was done in Kaleidagraph v 3.5 for a rough estimate of the parameters, and the curve plotting. Fine tuning of the curve fitting was done in SPSS v 12.0.1.

The dose-responses of the Cd and paraquat exposures on the RLU of every construct were compared by estimating the RLU_max_, EC_50 _and the slope by fitting a curve with the following formula, from which RLU_max _by summing RLU_0 _(the average RLU at unexposed conditions) and RLU_e_.

RLU=RLU0+RLUest1+e(−slope.ln⁡([Cd]/EC50))
 MathType@MTEF@5@5@+=feaafiart1ev1aaatCvAUfKttLearuWrP9MDH5MBPbIqV92AaeXatLxBI9gBaebbnrfifHhDYfgasaacH8akY=wiFfYdH8Gipec8Eeeu0xXdbba9frFj0=OqFfea0dXdd9vqai=hGuQ8kuc9pgc9s8qqaq=dirpe0xb9q8qiLsFr0=vr0=vr0dc8meaabaqaciaacaGaaeqabaqabeGadaaakeaacqWGsbGucqWGmbatcqWGvbqvcqGH9aqpcqWGsbGucqWGmbatcqWGvbqvdaWgaaWcbaGaeGimaadabeaakiabgUcaRmaalaaabaGaemOuaiLaemitaWKaemyvau1aaSbaaSqaaiabdwgaLjabdohaZjabdsha0bqabaaakeaacqaIXaqmcqGHRaWkcqWGLbqzdaahaaWcbeqaaiabcIcaOiabgkHiTiabdohaZjabdYgaSjabd+gaVjabdchaWjabdwgaLjabc6caUiGbcYgaSjabc6gaUjabcIcaOiabcUfaBjabdoeadjabdsgaKjabc2faDjabc+caViabdweafjabdoeadnaaBaaameaacqaI1aqncqaIWaamaeqaaSGaeiykaKIaeiykaKcaaaaaaaa@5AFA@

The 20-E exposure data were fitted with the following formula and RLU_min_, slope and EC_50 _were estimated.

RLU=RLUest+RLU01+e(slope.ln⁡([Cd]/EC50))
 MathType@MTEF@5@5@+=feaafiart1ev1aaatCvAUfKttLearuWrP9MDH5MBPbIqV92AaeXatLxBI9gBaebbnrfifHhDYfgasaacH8akY=wiFfYdH8Gipec8Eeeu0xXdbba9frFj0=OqFfea0dXdd9vqai=hGuQ8kuc9pgc9s8qqaq=dirpe0xb9q8qiLsFr0=vr0=vr0dc8meaabaqaciaacaGaaeqabaqabeGadaaakeaacqWGsbGucqWGmbatcqWGvbqvcqGH9aqpcqWGsbGucqWGmbatcqWGvbqvdaWgaaWcbaGaemyzauMaem4CamNaemiDaqhabeaakiabgUcaRmaalaaabaGaemOuaiLaemitaWKaemyvau1aaSbaaSqaaiabicdaWaqabaaakeaacqaIXaqmcqGHRaWkcqWGLbqzdaahaaWcbeqaaiabcIcaOiabdohaZjabdYgaSjabd+gaVjabdchaWjabdwgaLjabc6caUiGbcYgaSjabc6gaUjabcIcaOiabcUfaBjabdoeadjabdsgaKjabc2faDjabc+caViabdweafjabdoeadnaaBaaameaacqaI1aqncqaIWaamaeqaaSGaeiykaKIaeiykaKcaaaaaaaa@5A0D@

The RLU values from 20-E exposure concentrations at which a putative induction was observed were tested by a One Way ANOVA (p < 0.05) to test for induction.

## Authors' contributions

TJ performed the genome walking, cloning, sequencing, reporter assays, the data analysis and drafted the manuscript. JM and DR performed the initial universal fast walking and assisted in the field and lab work. JL and PC supported the cell culture and luciferase reporter assays and evaluated the manuscript. DR and NvS assisted in outlining the study, directing research and revision of the manuscript.

## Supplementary Material

Additional file 1 Alignment of the primary sequences. The primary sequences were aligned with the consensus sequences of the alleles identified in the *mt *coding sequence [26] Click here for file

Additional file 2 Alignment per allele. These alignments show the variation between the clones from which the consensus sequence for every allele was extracted. Click here for file

Additional file 3 Alignment per allele. These alignments show the variation between the clones from which the consensus sequence for every allele was extracted. Click here for file

Additional file 4 Alignment per allele. These alignments show the variation between the clones from which the consensus sequence for every allele was extracted. Click here for file

Additional file 5 Alignment per allele. These alignments show the variation between the clones from which the consensus sequence for every allele was extracted. Click here for file

Additional file 6 Alignment per allele. These alignments show the variation between the clones from which the consensus sequence for every allele was extracted. Click here for file

Additional file 7 Alignment per allele. These alignments show the variation between the clones from which the consensus sequence for every allele was extracted. Click here for file

Additional file 8 Alignment per allele. These alignments show the variation between the clones from which the consensus sequence for every allele was extracted. Click here for file

Additional file 9 Alignment per allele. These alignments show the variation between the clones from which the consensus sequence for every allele was extracted. Click here for file

Additional file 10 Alignment of the *pmt *alleles with *Orchesella villosa pmt *as an outgroup. These data were used in the phylogenetic and recombinational analysis. Click here for file

Additional file 11 Curve fit estimates of the luciferase reporter assay. Overview of the curve fit estimates for the exposure of every luciferase construct to Cd, paraquat and 20-E. Click here for file

Additional file 12 Primer Table. In this table the name and sequence of the used primers are given. Click here for file

Additional file 13 Sequence strategy Table. In this table the number and origin of the clones sequenced per allele are given. Click here for file
