# Supplementary material for: Recombinational micro-evolution of functionally different metallothionein promoter alleles from Orchesella cincta
Source: BMC Evol Biol. 2007 Jun 11;7:88. doi: 10.1186/1471-2148-7-88 (PMC1913499; doi:10.1186/1471-2148-7-88)
Supplement: Additional file 11 — Curve fit estimates of the luciferase reporter assay. Overview of the curve fit estimates for the exposure of every luciferase construct to Cd, paraquat and 20-E. [file 1471-2148-7-88-S11.doc]

|  | Cd | | | Paraquat | | | 20-E | | |
| --- | --- | --- | --- | --- | --- | --- | --- | --- | --- |
|  | RLUmax | Slope | EC50 (μM) | RLUmax | Slope | EC50  (μM) | RLUmin | Slope | EC50  (nM) |
| *pmt*A*luc* | 798  (701.-881) | 2.0 (1.4-2.5) | 6.2  (4.7-7.8) | 9.9  (9.1-10.7) | 5.6  (0.4- 10.7) | 640  (528-752) | 2.0  (1.2-2.7) | -9.0  (bad estimate) | 10.3  (bad estimate) |
| *pmt*B*luc* | 1283 (1130-1435) | 1.4 (1.2-1.5) | 11.1 (8.6- 13.6) | 13.3 (10.0-16.7) | 2.0 (-0.1-4.1) | 608 (158-1031) | 4.1  (2.9-5.3) | -1.2  (-2.6-0.1) | 15.0  (-0.1-30.1) |
| *pmt*C*luc* | 64 (47-81) | 1.8  (1.0- 2.5) | 9.7  (5.1-14.2) | No fit | No fit | No fit | 0.1  (0.0-0.2) | -0.3  (-0.5- -0.2) | 1000  (-1490.1- 3490.1) |
| *pmt*D1*luc* | 368 (306- 429) | 1.7  (1.2-2.2) | 7.3  (4.8-9.8) | 9.9 (8.1-11.8) | 1.5 (0.6-2.3) | 511 (247-774) | 1.3  (0.2-2.4) | -0.5  (-0.7- -0.3) | 23.9  (-6.7- 54.5) |
| *pmt*D2*luc* | 2434 (2220-2647) | 2,22 (1.8-2.7) | 9.9  (8.6-11.1) | 28.5  (-12.5- 69.4) | 1.3  (0.2-2.4) | 2059 (-3731-7849) | 1.7  (1.3-2.2) | -14.7  (bad estimate) | 89.2  (bad estimate) |
| *pmt*F*luc* | 3333  (2151-4516) | 1.2  (1.0- 1.5) | 22.1  (9.0-35.3) | 12.1 (10.7-13.5) | 2.7 (0.4-5.0) | 546 (370-723) | 2.3  (1.7-2.9) | -7.8  (bad estimate) | 77.2  (bad estimate) |
