# Supplementary material for: Recombinational micro-evolution of functionally different metallothionein promoter alleles from Orchesella cincta
Source: BMC Evol Biol. 2007 Jun 11;7:88. doi: 10.1186/1471-2148-7-88 (PMC1913499; doi:10.1186/1471-2148-7-88)
Supplement: Additional file 12 — Primer Table. In this table the name and sequence of the used primers are given. [file 1471-2148-7-88-S12.doc]

Table Primers and adapters used

| Primer | Sequence |
| --- | --- |
| Adapter1 | CTAATACGACTCACTATAGGGCTCGAGCGGCCGCCCGGGCAGGT |
| Adapter 1A | ACCTGCCC |
| Primer1 | CTAATACGACTCACTATAGGGC |
| Nested primer 1 | TCGAGCGGCCGCCCGGGCAGGT |
| R34 | GCACACGGCACTTTGCACGCTC |
| R130 | AGTGCCGTGTGCATGCATGTATATG |
| D1-36 FKpnI | AAAAGGTACCGGCATTCTAGTTTGAGTCAGTCTACCGG |
| MT-73RXhoI | GGGGAAACTCGAGTCGTTAGAGAATTTGAAGAACTGCG |
| D1-36 F | GGCATTCTAGTTTGAGTCAGTCTACCGG |
| MT265R | CCTTGCAGACACAATCTGGACC |
| F19 | TTTGCACACGGCACTTTGC |
| pmtFa | GCCGCTTTGTACCTGCGATG |
| pmtBALF | CGCTGGTACCACTGGTACCGTCC |
| pmtBALR | GCCAAAATTCTGCTTCTGCTTACATCC |
| pmtBALR2 | CTTACTGTGCATCAAGTGCTGTTGTTCC |
| Ovimt210R | CTCCATCCTTGCAAACGC |
| Ovimt233R | TGGACCACAGCAAGTGC |
| Ovipmt | CGCTTCCTACAAATACAATCACCAATTC |
| OvipmtF | GAATTGGTGATTGTATTTGTAGGAAGCG |
| OvipmtFa | CCGATCCATGAAACTTTCTATAGGCG |
| OvipmtfarF | GGCGAACAAAATGAATTTCCAGACG |
| OvipmtMF | GATTCGATCGATGAGAATATTCCCGC |
| OvipmtRnesed | CGCCTATAGAAAGTTTCATGGATCGG |
