# Supplementary material for: Recombinational micro-evolution of functionally different metallothionein promoter alleles from Orchesella cincta
Source: BMC Evol Biol. 2007 Jun 11;7:88. doi: 10.1186/1471-2148-7-88 (PMC1913499; doi:10.1186/1471-2148-7-88)
Supplement: Additional file 13 — Sequence strategy Table. In this table the number and origin of the clones sequenced per allele are given. [file 1471-2148-7-88-S13.DOC]

| *pmt* allele  consensus | Clones | | | |
| --- | --- | --- | --- | --- |
| *pmt*A1 | A10-9 **P** | C14-1 **C** | H12-11  H12-13 **P** | C24-2 **C** |
| *pmt*A2 | A2-4 **C** | B3-3  B3-14 **C** |  |  |
| *pmt*B | P8-5 **P** | G12-3 **P** | G8-3 **P** |  |
| *pmt*C | G8-2 **P** | G2-3 **C** | H1-1 **C**  H1-4 | D4-5 **C** |
| *pmt*D1 | P50-2 **P**  P50-7 | P62-5 **P** | A2-1 **C** | pmtSTOLC5-1 **S** |
| *pmt*D2 | G7-2 **P** | pmtMAKA4-8 **M** | pmtSTOLF3-4 **S** |  |
| *pmt*F | E7-8 **P** | X46-2 **C** | X16-2 **C** |  |
| *pmt*E | pmtABSB4-5 **A** | pmtHOBCC10-`1 **H** | pmtMAA1E5-8 pmtMAA1E5-12 **L** |  |
| *pmt*BAL | pmtBALA3-10  pmtBALA3-15 **B** |  |  |  |

Table: Overview of the clones sequenced to achieve the consensus sequence per allele. Clones from the same individual are located in the same field. C, Lab culture from Roggebotzand (NL) (clean forest on reclaimed land); P, Historical Pb and Zn mine, Plombières (B); S, Vicinity of non-ferro metal smelter, Stolberg (D); M, Beech forest, Makkegem (B); L, Vicinity of former non-ferro metal smelter, Lommel (B); B, Vicinity of non-ferro metal smelter, Balen (B). The underlined clones were used to construct the luciferase reporter vectors.
